# ABO blood grouping and COVID-19: a hospital-based study in Eastern India

**DOI:** 10.1186/s43042-022-00225-9

**Published:** 2022-01-20

**Authors:** Birasen Behera, Bidyutprava Rout, RajashreePanigrahy, Subrat Kumar Kar, Debasish Sahoo, Kundan Kumar Sahu, Sarita Otta

**Affiliations:** Department of Microbiology, IMS and SUM Hospital, Kalinga Nagar, Bhubaneswar, 751003 India

**Keywords:** COVID-19, ABO blood type, SARS-COV2, Real-time PCR

## Abstract

**Background:**

Blood group has been stated to be one of the risk factors associated with viral diseases like dengue, hepatitis virus, Norwalk virus and even the coronavirus associated with 2003 severe acute respiratory syndrome (SARS) outbreak. In addition, anti-A antibodies in experimental models have been shown to inhibit the interaction between coronavirus and angiotensin converting enzyme (ACE) receptor of the host target cell, the major receptor involved in viral pathogenesis. Thus, several workers propose an association between ABO blood type and coronavirus disease- 2019 (COVID-19) disease in many previous studies. The present study was undertaken in the Eastern part of India in line with these authors to study the association of ABO blood group of patients with COVID susceptibility and severity.

**Methods:**

This is a retrospective study over a period of 6 months from June 2020 to November 2020 where patients who underwent quantitative real-time polymerase chain reaction (qRT-PCR) test for SARS-COV2 and having a recorded patient blood group type were considered. The qRT-PCR positive admitted cases were considered as cases, and qRT-PCR negative cases were considered as controls. Data were entered in Microsoft Excel format and analyzed by statistical method to obtain association.

**Results:**

Consecutively obtained 5000 qRT-PCR positive patients (cases) and 11,700 (controls) were included in the present study. The mean age of cases was higher (54.24 vs. 34. 67) than the controls. Among the cases, the highest number (2379; 47.6%) of samples belonged to A blood group followed by B (1278; 25.6%) while among the control group O blood group had the highest prevalence (4215; 36%). Blood group A had a higher odd of testing positive (Odds ratio-2.552; CI 2.381–2.734; *p* < 0.0001) than all other blood groups. A blood group is also associated with higher risk of ICU admission (Odds ratio- 1.699; 95% CI 1.515–1.905) and 65.3% of this group is also associated with high viral load which gives an indication of higher disease severity.

**Conclusion:**

Blood group A is associated with an increased susceptibility to COVID 19 infection than other blood groups. Cases of this blood group are also associated with more critical care needs and a higher viral load on testing.

## Background

Since the declaration of COVID 19 (Coronavirus Disease -2019) as a pandemic by World Health Organization, it has created havoc worldwide with huge surge in case load and thus mortality and morbidity. There is yet no biomarker developed to detect the susceptibility of the patients to the COVID 19 disease [1]. Risk factors proposed till date are old age, male sex and chronic diseases like diabetes, hypertension and cardiovascular diseases [2]. The ABO blood group antigens are glycoproteins present mostly on surface of erythrocytes and are encoded by co-dominant alleles (A and B) present in Chromosome 9 [3]. Blood group expression varies greatly among different races and it has been associated as a genetic risk factor for different infectious diseases and malignancies like Hepatitis B virus, Norwalk virus, Dengue virus and even the last corona viral SARS (severe acute respiratory illness) outbreak in 2003 [4]. Anti-A antibodies inhibit the interaction between severe acute respiratory syndrome coronavirus 2 (SARS-COV 2) and Angiotensin Converting Enzyme (ACE) receptor expressed on host target cells. ACE receptors being the major target of this virus it is logical to expect an association between the ABO blood group and COVID 19 disease. Li et al. [5] was the foremost researcher to show that blood group A has a significantly higher risk of SARS-CoV2 infection and blood group O has a lower risk. Several other works since then have associated COVID-19 and ABO blood group [3, 6–9]. In continuation of these works, the present study aims to probe into association between ABO blood group and SARS-CoV-2 infection in Eastern part of India.

## Methods

### Study setting

The present study was conducted at IMS and SUM Hospital a tertiary care hospital with dedicated quantitative real-time polymerase chain reaction (qRT-PCR) laboratory for testing as well as wards and ICU for COVID 19 patient care. This is a retrospective study conducted over a period of 6 months from June 2020 to November 2020 where data obtained from the Hospital Information System were collected.

### Method

Nasopharyngeal and oropharyngeal swabs collected from patients in viral transport medium were used for detection of SARS COV2 by qRT-PCR technique. RNA was extracted using In vitrogen kit, and qRT-PCR was performed using Taq Path kit (Thermo Fisher Scientific) on ABI 7500-Fast, Thermo-fisher machine. The cycle threshold (Ct) value for each sample was recorded. We have collected the relevant patient admission details, laboratory parameters including blood grouping and patient disease severity from the hospital information system.

### Study population

The patients, who were admitted to the hospital after testing as SARS-COV2 positive by qRT-PCR for symptoms requiring the said testing, were denoted as cases. (Inclusion criteria) Any other patient who presented to the hospital with symptoms which mandated COVID-19 testing as decided by the clinician but were negative by the qRT-PCR were denoted as controls for the present study. The patients of all age, gender and blood type were included in this study.

### Exclusion criteria

The patients for whom we could not find relevant information in hospital system, those who were not admitted in this hospital among qRT-PCR positive group or those treated in outpatient basis after obtaining a positive result following testing were excluded from the study.

### Statistical analysis

Data were entered in excel sheet and analysis done using standard statistical methods. Statistical analysis was performed using Graph Pad Prism 7. Data were analyzed with the *χ*^2^ test and Fisher's exact test to note the distribution of blood groups. Odds Ratio with 95% Confidence Interval for all the blood types in cases were assessed with logistic regression models. Probability value (*p*) < 0.05 was considered statistically significant.

### Ethical consideration

All the samples collected for testing were received by the laboratory after a receiving an implied consent from the patient for diagnosis or treatment purpose in this hospital. During the tenure of the study, no individual history was disclosed in any form. This study was approved by institutional ethical committee via Ref.no/DRI/IMS.SH/ SOA/2021/174 dated 31st August 2021.The IEC is registered by registration number- ECR/627/Inst/OR/2014/RR-20.

## Results

### Distribution of cases and controls in the study as per age and sex

**Fig. 1:**
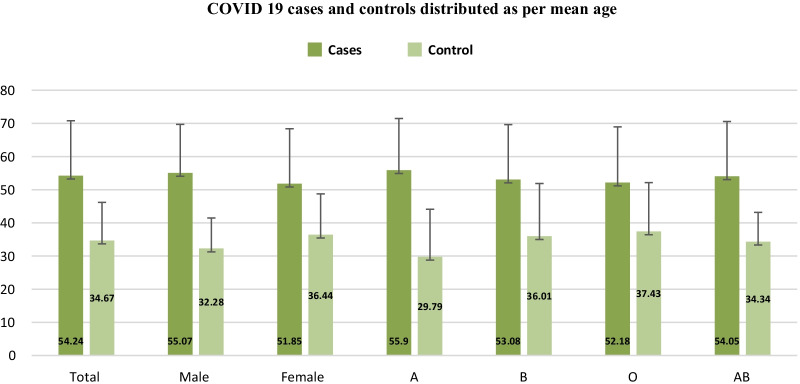
COVID 19 cases and controls distributed as per mean age

A total of 16,700 patients were consecutively enrolled in the present study, among whom 5000 patients were found as SARS-COV2 qRT-PCR positive (cases) and 11,700 patients had SARS-COV2 qRT-PCR negative result (controls). Of the 5000 cases, highest number of samples belonged to 51–60 age group. The mean age of cases was much higher (54.24 vs. 34. 67) than the controls, the difference manifesting across both the sexes (Males—55.07 vs. 32.28; Females—51.85 vs. 36.44) as well as across all the blood groups (Fig. 1). The mean and standard deviation of patients admitted in ICU and wards in this study were 55.83 ± 15.03 and 51.65 ± 16.11, respectively. Ratio of males to female was 2.9:1 in this study.

### Analysis of susceptibility to COVID-19 infection

Among the cases, the highest number of samples belonged to blood group A (2379; 47.6%) followed by B (1278; 25.6%) and O (1174; 23.5%). On the other hand, among the control group, O blood group had the highest prevalence (4215; 36%) followed by B blood group (3926; 33.5%), A (3070; 26.2%), AB (489; 4.12%). The difference in susceptibility of cases and controls to COVID 19 among A, B, O blood group was significant at *p* < 0.05 while there is no significant difference in susceptibility in AB blood group (Table 1).

**Table 1:** Distribution of cases and controls as per their blood types

Blood Group	Number of cases (% total)	Number of controls (% total)	*χ*^2^	*p* value
A	2379 (47.6)	3070 (26.2)	26.94	< 0.0001
B	1278 (25.6)	3926 (33.5)	10.22	< 0.0001
O	1174 (23.5)	4215 (36.0)	15.88	< 0.0001
AB	169 (3.4)	489 (4.12)	2.43	< 0.0150
Total	5000	11,700		

Blood group A had a higher odd of testing positive than all other blood groups. It is associated with increased risk of infection (Odds ratio-2.552; CI 2.381–2.734; *p* < 0.0001) while all other blood group showed a decrease in risk (Table 2).

**Table 2:** Comparison of ABO blood group distributions

Blood group	Odds ratio (95% confidence interval)	*χ*^2^	*p* value
A-B	1.999 (1.838–2.173)	16.28	< 0.0001
A-O	2.781 (2.557–3.025)	24.26	< 0.0001
A-AB	2.238(1.864–2.687)	8.83	< 0.0001
A-Non-A	2.552 (2.381–2.734)	26.94	< 0.0001
B-Non-B	0.680 (0.635–1.584)	10.22	< 0.0001
O-Non-O	0.545 (0.505–0.588)	15.88	< 0.0001
AB-Non-AB	0.802 (0.672–0.958)	2.43	< 0.015

### Analysis of severity of COVID-19 infection

Among the patients included in the study period, 2806 (56.12%) patients were admitted in ICU while 2194 (43.9%) patients were in wards. The cases of A, B and O were more commonly admitted to ICU than wards with significant *p* value of < 0.00001. On the other hand, AB blood group had no significant difference in admission of cases to ICU or wards. Blood group A is associated with higher risk of ICU admission (Odds ratio- 1.699; 95% CI 1.515–1.905) while all other blood groups have a lower risk (Table 3).

**Table 3:** Distribution of cases as per their admission status

Blood group	Number of patient in Wards (% of total)	Number of patients in ICU(% total)	*χ*^2^ value	*p* value	Odds ratio (95% CI)
A	806 (33.9)	1573 (66.1)	9.112	< 0.0001	1.699 (1.515–1.905)
B	600 (46.9)	678 (53.1)	5.426	< 0.0001	0.702 (0.617–0.798)
O	551 (46.9)	623 (53.1)	5.117	< 0.0001	0.709 (0.622–0.809)
AB	69 (40.8)	100 (59.2)	0.083	= 0.934	0.987(0.723–1.353)
Total	2026 (40.5)	2974 (59.5)			

**Fig. 2:**
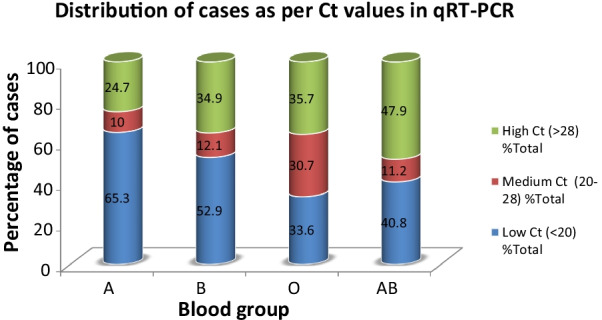
Distribution of cases as per their Ct values in qRT-PCR for SARS-COV2

The patients were further divided as per the Ct value which is an indirect measure of viral load. More than half of cases (53.9%) were having low Ct value denoting a high viral load; moderate Ct value (20–28) had the minimum number (15.4%) of cases. Blood groups A, B showed a higher proportion of cases in low Ct group; blood group A showing the highest percentage (1553, 65.3%). On the other hand, O and AB blood groups had higher percentage of samples from high Ct (Fig. 2).

## Discussion

The COVID-19 pandemic has till now grasped 190,597,409 confirmed cases all over the world including 4093,145 deaths and numbers are rising daily [10]. Knowing the important risk factors come in handy to identify the at risk population, to add new precautions and judicious allocation of the available resources to prevent the wide spread infection and mortality. There is a dearth of knowledge on demographic and clinical risk element that controls the susceptibility to SARS CoV-2 infection and mortality. The risk factors identified till date include age, sex, few chronic conditions and laboratory findings. [11].

ABO gene is highly pleomorphic and differs widely across geographies and ancestry. This contains A and B antigens on red cell surface encoded by two dominant and two recessive alleles located on chromosome 9q34.1–34.2 [12]. These antigens are also expressed on epithelial cells, platelets, vascular endothelial cells and neurons [13]. ABO antibodies are part of the innate immune system against parasites, bacteria and enveloped viruses, and these also act as receptors for many immune and inflammatory responses. [4, 14].

In our study, mean age of cases was higher than the controls (54.24 vs. 34. 67). Most of our cases belonged to age group 51–60. In other similar studies [3, 15, 16], age has been defined as a risk factor as well most common age group being the fifties. This is probably due to the fact that this is the age group of common predominance of several chronic illnesses as well as the natural immunological detritions associated with aging. In the present study, males outnumbered females like in previous similar studies. [3]

In this study, all of the blood groups except AB (A, B, and O) showed a significant difference between cases and controls. Blood group A is associated with increased risk of infection (Odds ratio-2.552; CI 2.381–2.734; *p* < 0.0001). Blood group A constituted the highest percentage of cases while in controls blood group O was the commonest, which may suggest a protective effect of O phenotype. Many similar studies also found highest prevalence of A blood group in COVID 19 disease [5, 12, 17, 18]. This finding is again consistent with coronavirus 2003 SARS outbreak data [19]. coronavirus receptor binds to ACE2 receptor [20] and anti-A antibody inhibits the interaction between SARS-CoV-2 and the ACE2 receptor [21]. This is the proposed mechanism by which ABO blood groups influence SARS-CoV-2 infection and COVID-19 disease severity [5, 12, 18]. A study has also found that people with blood group O are able to recognize certain proteins like the viral surface antigens as foreign and so less likely to get a disease [22]. Studies by Padhi et al. [9], Latz et al. [23], Almadhi et al. [24] and Liu et al. [25] across continents report blood type B as having higher risk of testing positive for SARS-CoV-2 infection contradicting our finding. Further known prothrombotic effect of non-O blood group may also be playing a role [26]. Different in results in these studies indicate other unexplored factors.

Assessment of severity in these patients was done in the present study by using their rate of admission to ICU for critical care support as well as Ct values as an indirect marker of the viral load. High viral load as denoted by low Ct value is one of the poor prognostic factors. In the present study, 65.3% of the blood group A patients were having high load of viruses in the body. Similarly, the rate of admission in ICU was significantly higher *p* < 0.001) in A, B, O blood groups. Blood group A cases had a significant risk of ICU admission (Odds ratio-1.699) while all other blood groups had a negative risk. In a similar study by Latz et al. [23] A blood group had highest risk of intubation and death. Another study proposed that individuals with B blood group had higher risk of intubation, but this group was associated with lower mortality than blood group O [18]. On the other hand, blood group O was found to be associated with lower mortality in a study [23]. The association of ABO blood group and COVID-19 severity may involve other factors like specific anti-A titers [27], the immunoglobulin isotype of anti-A antibodies [28], and ABO group differences in von Willebrand factor which need further clarification. [29].

## Conclusions

Blood group A patients are more susceptible to COVID-19 in our subset. This blood group also has a significantly higher chance of critical care need as well as higher viral load. However, more studies with larger sampling particularly in community setting with an eye to nullify the effect of co-morbid conditions are necessary to confirm these findings. The mechanism of interaction between the ABO blood groups and ACE2 that forms the basis of this finding needs to be further investigated.

## Data Availability

Available with corresponding author.

## Abbreviations

ACE: Angiotensin converting enzyme
COVID-19: Coronavirus Disease -2019
CI: Confidence interval
Ct: Cycle threshold
ICU: Intensive care unit
OR: Odds ratio
*p*Value: Probability value
qRT-PCR: Quantitative real-time polymerase chain reaction
RNA: Ribonucleic acid
SARS: Severe acute respiratory syndrome
SARS- COV2: Severe acute respiratory syndrome coronavirus 2
